# Purification of linearized template plasmid DNA decreases double-stranded RNA formation during IVT reaction

**DOI:** 10.3389/fmolb.2023.1248511

**Published:** 2023-09-29

**Authors:** Juan Martínez, Verónica Lampaya, Ana Larraga, Héctor Magallón, Diego Casabona

**Affiliations:** RNA Synthesis and Development Department, Certest Pharma, Certest Biotec, Zaragoza, Spain

**Keywords:** dsRNA, chromatography, polishing, IVT, immunogenicity, vaccines, mRNA

## Abstract

After the COVID-19 pandemic, messenger RNA (mRNA) has revolutionized traditional vaccine manufacturing. With the increasing number of RNA-based therapeutics, valuable new scientific insights into these molecules have emerged. One fascinating area of study is the formation of double-stranded RNA (dsRNA) during *in vitro* transcription (IVT) which is considered a significant impurity, as it has been identified as a major trigger in the cellular immune response pathway. Therefore, there is a growing importance placed to develop and optimize purification processes for the removal of this by-product. Traditionally, efforts have primarily focused on mRNA purification after IVT through chromatographic separations, with anion exchange and reverse phase chromatography emerging as effective tools for this purpose. However, to the best of our knowledge, the influence and significance of the quality of the linearized plasmid have not been thoroughly investigated. Plasmids production involves the growth of bacterial cultures, bacterial harvesting and lysis, and multiple filtration steps for plasmid DNA purification. The inherent complexity of these molecules, along with the multitude of purification steps involved in their processing, including the subsequent linearization and the less-developed purification techniques for linearized plasmids, often result in inconsistent batches with limited control over by-products such as dsRNA. This study aims to demonstrate how the purification process employed for linearized plasmids can impact the formation of dsRNA. Several techniques for the purification of linearized plasmids based on both, resin filtration and chromatographic separations, have been studied. As a result of that, we have optimized a chromatographic method for purifying linearized plasmids using monolithic columns with C4 chemistry (butyl chains located in the surface of the particles), which has proven successful for mRNAs of various sizes. This chromatographic separation facilitates the generation of homogeneous linearized plasmids, leading to mRNA batches with lower levels of dsRNA during subsequent IVT processes. This finding reveals that dsRNA formation is influenced not only by RNA polymerase and IVT conditions but also by the quality of the linearized template. The results suggest that plasmid impurities may contribute to the production of dsRNA by providing additional templates that can be transcribed into sequences that anneal with the mRNA molecules. This highlights the importance of considering the quality of plasmid purification in relation to dsRNA generation during transcription. Further investigation is needed to fully understand the mechanisms and implications of plasmid-derived dsRNA. This discovery could shift the focus in mRNA vaccine production, placing more emphasis on the purification of linearized plasmids and potentially saving, in some instances, a purification step for mRNA following IVT.

## 1 Introduction

In recent years, mRNA-based therapies have emerged as highly promising tools in combating infectious diseases, correcting malfunctioning genes, and offering new approaches to tackle pathogens and tumors. The SARS-CoV-2 pandemic has served as a catalyst, propelling advancements in this technology and necessitating the development of production techniques with stringent timelines to address a global health crisis. (Schlake et al., 2012; Kis et al., 2020; Whitley et al., 2020; Ouranidis et al., 2021; Park et al., 2021; Sousa et al., 2021; Verbeke et al., 2021).

Due to the urgent need for mRNA production, IVT processes were developed at a rapid pace, often surpassing the understanding of the molecule itself and the by-products that arise during the transcription process. The transcription of DNA templates into mRNA is facilitated by phage RNA polymerases, such as T7 RNA polymerase (T7 RNAP), which exhibit high fidelity in RNA synthesis. However, the biological process is not entirely efficient, resulting in the generation of certain by-products, albeit at a low frequency. Common non-target molecules include abortive sequences, short transcripts, and dsRNA. (Mu and Hur, 2021).

From the above described, dsRNA moieties have special interest due to inherent immunogenicity that is not desirable in mRNA therapeutic applications. The innate cellular response to dsRNA byproducts, recognized by receptors like TLR3, can adversely affect mRNA therapy efficacy. TLR3 activation by dsRNA involves multiple steps, including dimerization and activation upon dsRNA recognition. This recruits cytoplasmic adapter TRIF, initiating downstream signaling with adaptors like TRAF3 and RIP1. This leads to intracellular signaling events, activating transcription factors IRF3 and NF-κB, which promote immune gene transcription. Additionally, a Src-mediated, adapter-independent TLR3 branch influences cellular properties. In summary, TLR3 activation by dsRNA initiates a complex cascade involving TRIF and transcription factors, inducing immune genes. Src’s adapter-independent branch modulates cellular properties beyond gene induction (Mu and Hur, 2021).

Origin of this double stranded molecule is being studied and is not very well stablished yet. Most of the authors described a great dependence of the T7 RNAP enzyme. It has been described during the initiation of transcription; 5- to 11-nt-long RNA by-products are generated as the enzyme aborts the synthesis with a certain probability (Milligan et al., 1987; Dousis et al., 2022).

**FIGURE 1:**
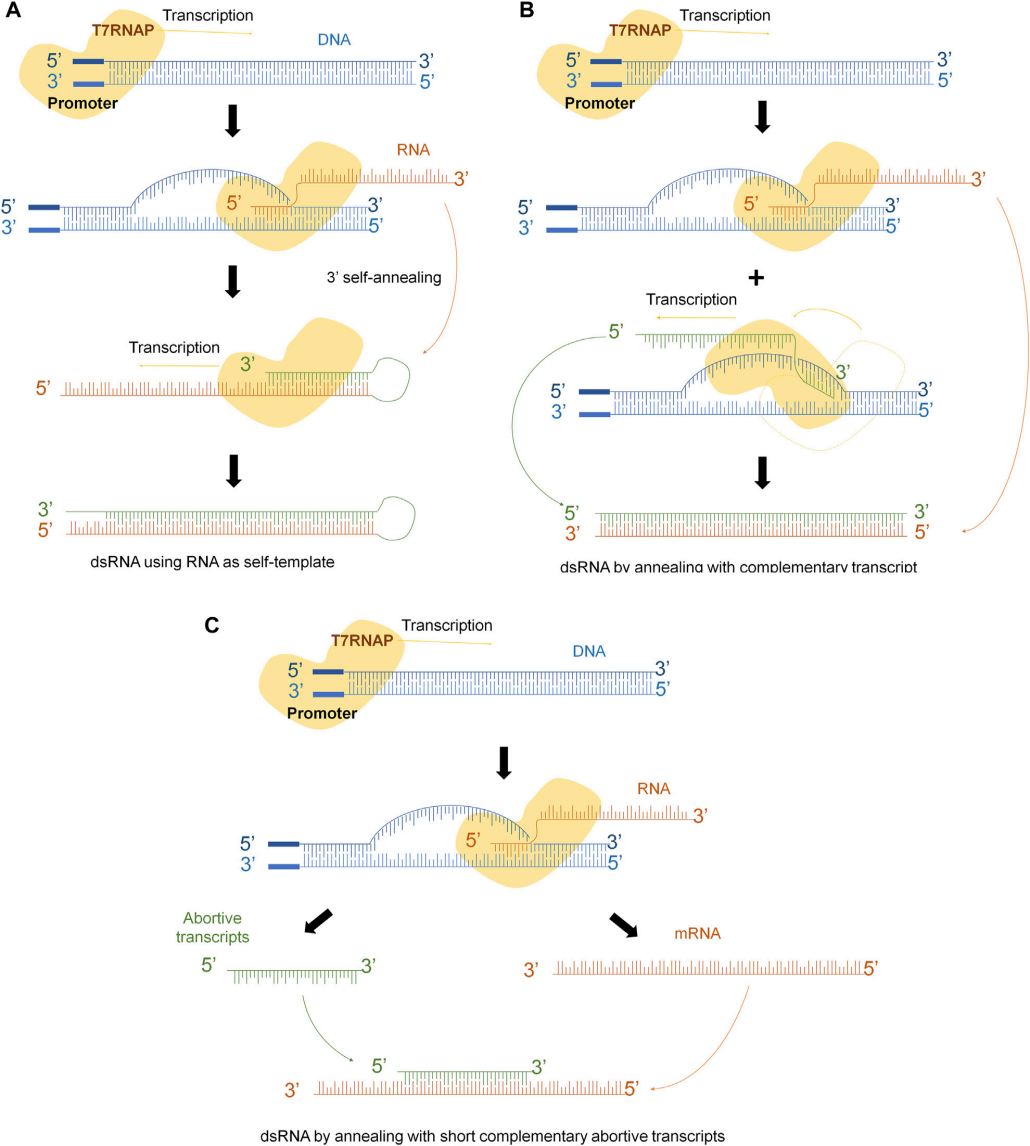
dsRNA generation mechanisms during IVT. **(A)** Template strand of DNA is transcribed into mRNA, 3’-extreme loops due to complementarity and T7RNAP uses mRNA as template to continue transcription. **(B)** Polymerase might switch to the non-template strand and generate the complementary strand of the original mRNA. **(C)** Abortive fragments during IVT with complementary sequences anneals with the run-off transcript.

Several mechanisms have been described to explain the different pathways that lead to these kinds of molecules. One of them, explains that RNA transcript that has been synthesized by the T7 RNAP serves as a template for the RNA-dependent RNA polymerase activity of the T7 RNAP in subsequent rounds of transcription (Roy and Wu, 2019). If the 3′-end of the runoff transcript has sufficient complementarity (in cis), it will fold back and result in extension of the runoff transcript (Figure 1A). Another explanation claims that T7 RNAP might use the resulting RNA molecule as template to synthesize the complementary strand in a promoter-independent manner (Figure 1B) (Triana-Alonso et al., 1995; Gholamalipour et al., 2018). Another mechanism considers that T7 RNAP also has subjacent RNA-dependent as well as template-independent RNA polymerase activity (Konarska and Sharp, 1989; Krupp, 1989; Cazenave and Uhlenbeck, 1994; Triana-Alonso et al., 1995; Biebricher and Luce, 1996; Arnaud-Barbe, 1998; Nacheva and Berzal-Herranz, 2003; Gholamalipour et al., 2018). Indeed, the short abortive RNA fragments and the 3‘-end of the full-length RNA can prime complementary RNA synthesis from the primary transcripts that leads to the generation of dsRNA contaminant (Figure 1C). However, it is crucial to acknowledge that dsRNA is not a precisely defined individual molecule, but rather a diverse population of molecules with varying sizes and annealing levels.

The permissible thresholds of dsRNA content exhibit variability contingent upon the designated application, whether within investigative, preclinical, or clinical contexts. Furthermore, these thresholds are inherently contingent upon the inherent characteristics of the mRNA in question. Presently, regulatory entities are diligently engaged in the endeavor to attain a harmonious consensus regarding the precise delineation of specifications governing mRNA vaccines. In accordance with prevailing conventions, it is widely acknowledged that dsRNA content should ideally remain below 0.5% for applications within clinical settings. However, the attainment of such modest dsRNA levels may prove to be a formidable undertaking through mere refinement of IVT conditions. As such, the implementation of supplementary purification protocols invariably becomes imperative to surmount this challenge effectively.

The presence of dsRNA impurities, which share similar physicochemical properties with single-stranded RNA (ssRNA) molecules, poses challenges in their removal during purification processes. Conventional purification methods such as lithium chloride precipitation, size exclusion chromatography, affinity chromatography, or diafiltration are not effective in eliminating dsRNA moieties. Anion exchange chromatography has emerged as good option for dsRNA removal in mRNA polishing (Levanova and Poranen, 2018; Baiersdörfer et al., 2019; Romanovskaya et al., 2013). However, scaling up these chromatographic purifications for industrial production is problematic. Recently, cellulose-based matrix purification methods have been proposed as suitable and simple approaches for dsRNA removal, irrespective of length and nucleoside composition (Baiersdörfer et al., 2019). Another strategy focuses on optimizing the transcription reaction conditions to minimize the production of dsRNA contaminants during IVT instead of relying solely on additional purification steps. Parameters such as reducing Mg2+ concentration (below 10 mM), using modified nucleosides, employing thermally stable T7 RNAP or mutated T7 RNAP, sequence optimization with depletion of U-rich sequences, or RNAse III treatment have been explored to address this issue (Wu et al., 2020; Cavac et al., 2021). It is important to note that the DNA template used in IVT also plays a crucial role, and the generation of dsRNA by-products could be influenced by the quality of the linearized DNA template in addition to the transcription enzyme.

In this study, we have successfully produced a wide range of constructs encompassing different sizes and scales. Additionally, we have thoroughly investigated various reaction conditions and purification methodologies. Commercially available plasmids are manufactured to meet specific customer requirements, and therefore, it is advisable to assess their compliance with specifications and determine the isoform status before commencing work with a new plasmid. The quality of plasmids directly impacts the quality of the resulting linearized template following the digestion step. Throughout our development process, we have observed significant heterogeneity in linearized plasmids, which appears to be dependent on the specific plasmid used. Interestingly, we have discovered a noteworthy correlation between the quality of linearized plasmids and the production of dsRNA. To the best of our knowledge, this study represents the first evidence linking plasmid quality to dsRNA generation during IVT.

## 2 Results

One of the primary objectives of optimization is to obtain RNA constructs of exceptionally high quality, with a specific focus on reducing levels of dsRNA. Initially, our efforts were directed towards the purification and refinement of mRNA, as many RNA producers and researchers have done in recent years.(Dickman and Hornby, 2006; Jacoby, 2006; Mccarthy and Gilar, 2008; Karikó et al., 2011; Weissman et al., 2013; Azarani and Hecker, 2021).

Various purification methodologies were combined for both linearized templates and mRNA to determine the optimal combination that would result in the lowest levels of dsRNA. Additionally, considering that dsRNA generation may be influenced by the length of the RNA, we investigated the impact of different construct sizes encoding three different proteins: Green Fluorescence Protein (GFP, 1,000 NTPs long aprox.), Firefly Luciferase (FLuc, 2000 NTPs long aprox.), and the Spike protein for SARS-CoV-2 (COV, 4,000 NTPs long aprox.). To minimize variables in the study and due to therapeutic mRNAs commonly contains modified nucleosides, we chose to employ N1-Me-ψUTP as the modified UTP for the production of all mRNA in this study (Knezevic et al., 2021; Kim et al., 2022).

**FIGURE 2:**
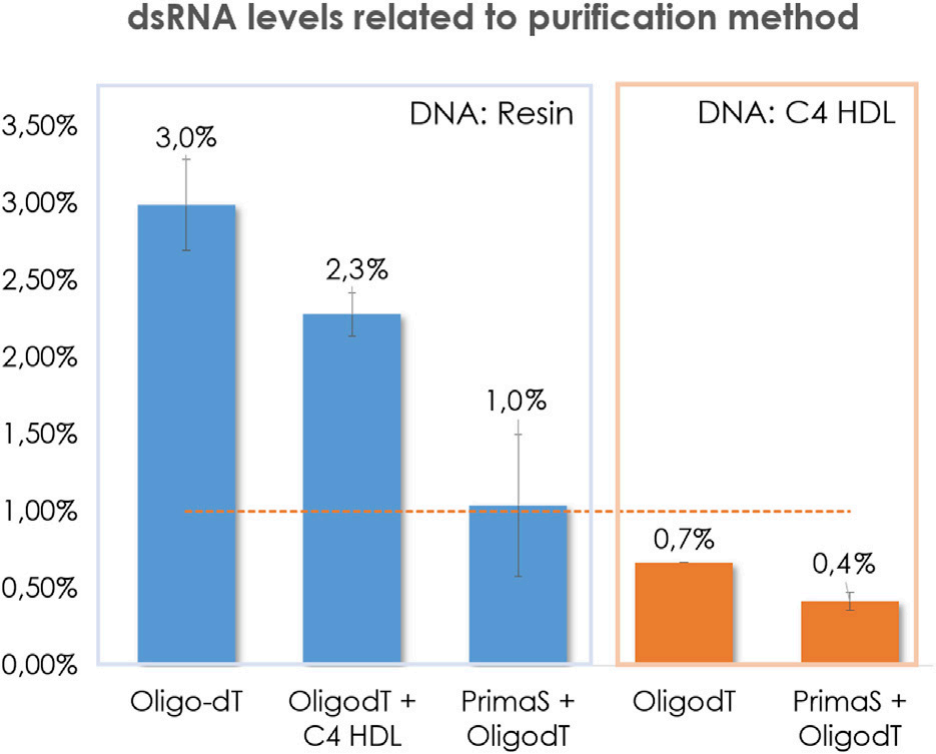
Comparison of the influence of the template purification method in the content of dsRNA in COVID transcripts. Pointed line represents threshold 1%.

At the first stage, our purification efforts were focused on mRNA that encoded COVID spike protein. After plasmid linearization, IVT crudes were purified by affinity chromatography (Oligo-dT) and levels of dsRNA were measured. It was founded that dsRNA contents were always above 2% and that most of them were higher than 3% (Figure 2). These impurity contents are unacceptable for therapy application, so it was mandatory to set up an additional step of polishing to reduce these amounts.

**FIGURE 3:**
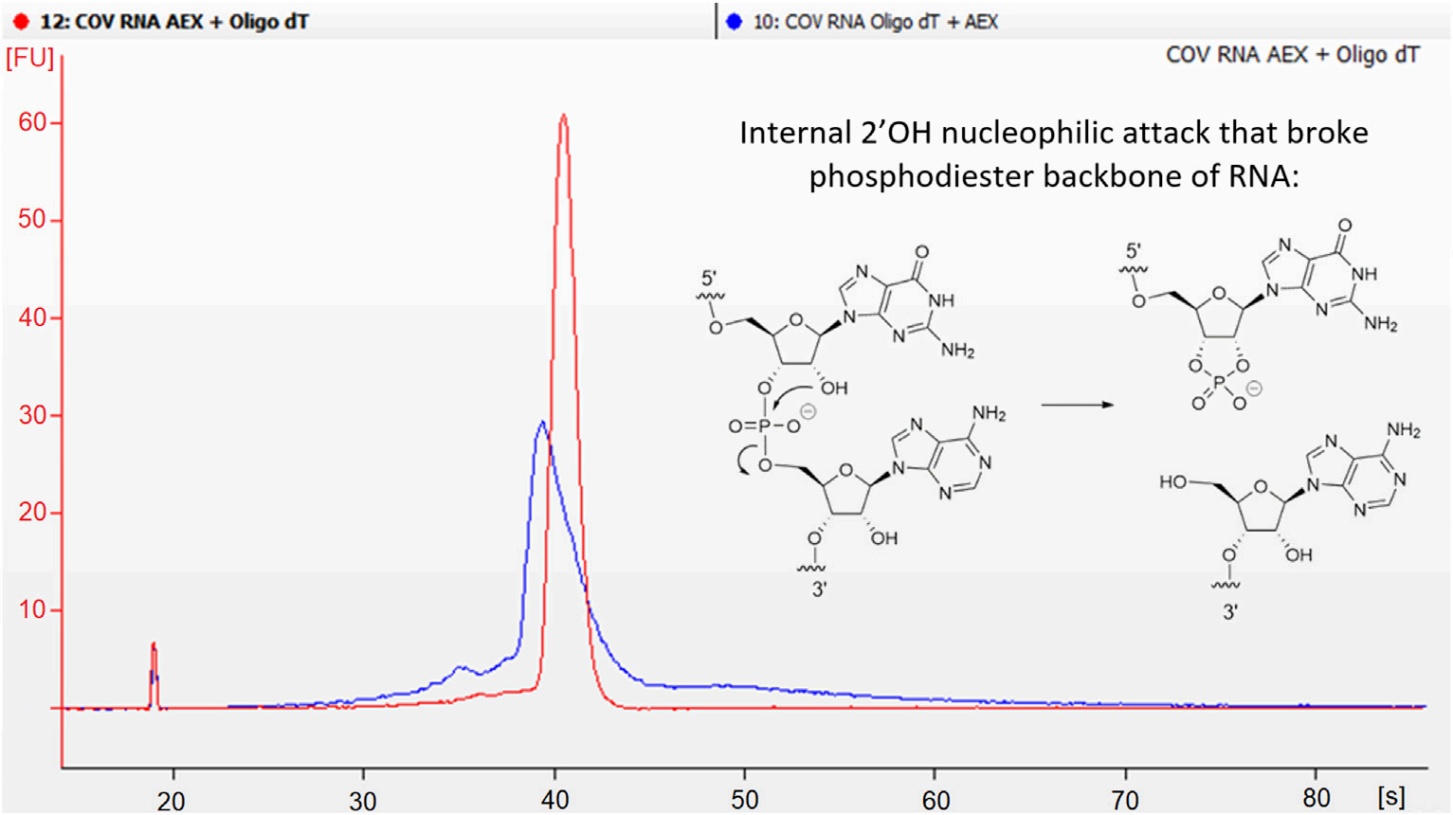
Degradation of mRNA is influenced by the type of purification method employed. Electropherogram comparison between tandem Oligo-dT + AEX (blue line) vs. AEX + Oligo-dT (red line). Blue line shows more degraded profiles. Mechanism of intramolecular hydrolysis of RNA phosphodiester bond.

On this regard, it was deemed interesting to compare dsRNA levels when an additional specific dsRNA removing polishing step was added after the DNA resin purification. Typically, tandem affinity (capture step) and anion exchange chromatography (polishing step) is very well known and accepted as a good path to obtain mRNA constructs with low levels of dsRNA and high purity (Gagnon, 2020). It is important to point out, that separation of dsRNA in AEX is usually performed by pH gradient, from 7.2 to 11. It is widely recognized that basic pH enhances RNA degradation by means of internal nucleophilic attack of 2’ hydroxyl to the nearest electrophilic phosphorus breaking the phosphodiester bond (Chatterjee et al., 2022; Fang et al., 2022). For this reason, the prompt neutralization of the collected fractions becomes crucially important. However, despite these efforts, a certain degree of degradation was observed. In an attempt to mitigate this degradation during our research, it was discovered that by reversing the order of these steps, improvements in purity were achieved as a result of reduced degradation. (Figure 3).

In order to prevent the loss of integrity, the decision was made to reverse the order of the purification process, starting with anion exchange chromatography (AEX) followed by affinity chromatography. Another commonly employed polishing strategy involves incorporating hydrophobic interaction chromatography (HIC) after affinity purification. Consequently, two additional polishing methods were employed: anion exchange chromatography using either the CIMmultus^®^ PrimaS^®^ 1 mL Monolithic Column (2 μm) or the CIMmultus^®^ C4HLD - 1 mL (2 µm). Both methods have been recognized for their effectiveness in removing dsRNA (Dickman and Hornby, 2006; Jacoby, 2006; Mccarthy and Gilar, 2008; Karikó et al., 2011; Weissman et al., 2013; Gagnon, 2020; Azarani and Hecker, 2021) (Urbas, 2015) (Mccarthy and Gilar, 2008; Podgornik et al., 2013; Lee and Barton, 2015; Separations, 2020).

By implementing the purification and polishing steps as mentioned earlier, we were able to reduce degradation in the final isolated constructs (Figure 2). As anticipated, the additional AEX step resulted in lower levels of dsRNA, with some cases falling below the 1% threshold, thus confirming its efficacy in removing such impurities. However, it was quite surprising to find that HIC was not as effective as AEX in reducing dsRNA levels.

### 2.1 Influence of purification of linear plasmids in dsRNA content

At this juncture, it is pertinent to recall that the prevailing methodologies employed for dsRNA removal primarily focus on the purification and polishing of mRNA post IVT, often overlooking the significance of purifying the initial linearized plasmid. However, we emphasize the importance of obtaining linear templates with high purity and quality, as they directly impact the levels of dsRNA. In order to investigate this, several batches of linear plasmids were isolated using a resin filtration method employing silica resin (Wizard^®^ Megapreps DNA Purification Resin, Promega, Cat#A7361) or by hydrophobic chromatography (CIMmultus^®^ C4HLD—1 mL (2 µm), Sartorius, Cat# 311.8136-2). Then IVT was carried out and the resulting crudes were purified and polished in some cases by Affinity (Oligo-dT) and AEX.

Surprisingly, the linearized plasmids isolated through chromatographic purification exhibited dsRNA levels below the 1% threshold. As anticipated, the subsequent polishing steps further reduced the presence of dsRNA. However, the tandem Oligo-dT/C4 HIC method was not as effective as the Prima S Oligo-dT method in removing double-stranded impurities, as demonstrated in Figure 2. These findings highlight the significant impact of template purification, which appears to have more influence than previously acknowledged.

These results suggest that based on a good purification method of the starting linearized plasmids, such as hydrophobic chromatography, it is possible to obtain mRNA transcripts that does not require any further polishing step to obtain less percentage of dsRNA.

## 3 Discussion

**FIGURE 4:**
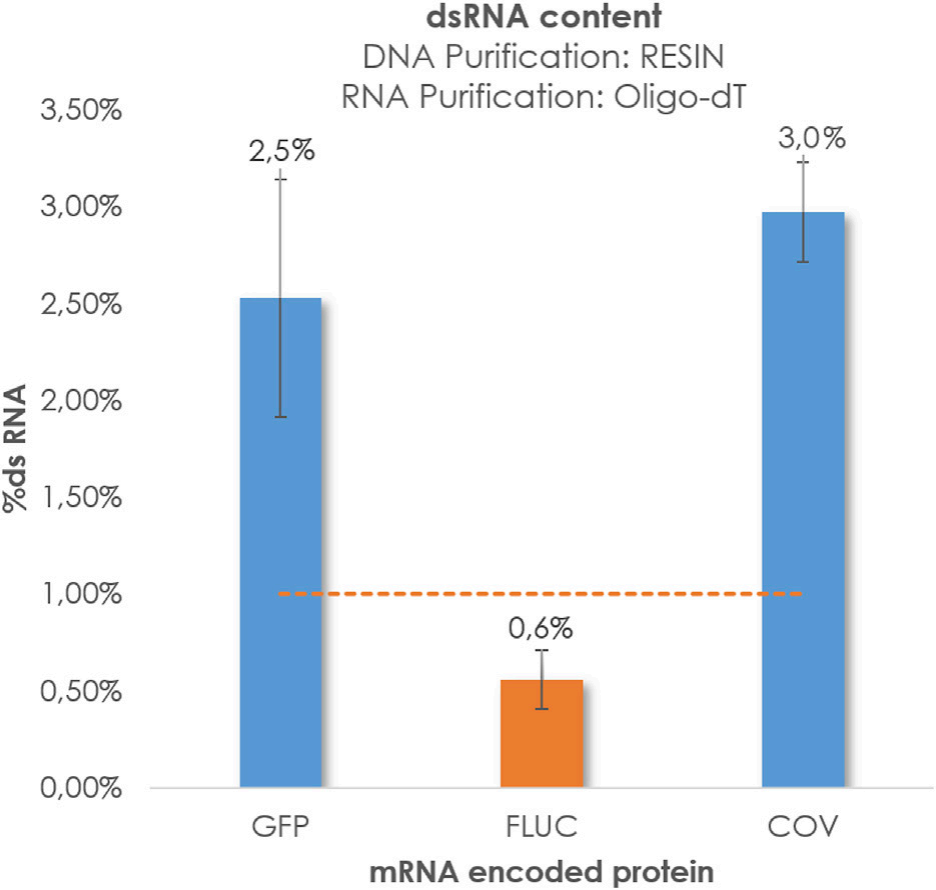
%dsRNA obtained for GFP, Fluc and COV. Linearized DNA was isolated by Wizard^®^ Megapreps DNA Purification Resin, Promega, Cat#A7361 and RNA crudes were purified by POROS™ Oligo (dT)25 Affinity column. Pointed line represents threshold 1%.

The primary objective of this study was to optimize our internal purification methods in order to reduce the presence of dsRNA and enhance the quality of the mRNA. As mentioned previously, significant efforts were dedicated to the polishing step following IVT. Until now, the generation of dsRNA has been largely attributed to the transcription process itself, with a substantial reliance on the polymerase enzyme. (Konarska and Sharp, 1989; Krupp, 1989; Cazenave and Uhlenbeck, 1994; Triana-Alonso et al., 1995; Biebricher and Luce, 1996; Arnaud-Barbe, 1998; Nacheva and Berzal-Herranz, 2003; Gholamalipour et al., 2018). According to this theory, it is suggested that pre-IVT processes, such as plasmid linearization, do not have an impact on dsRNA levels Nevertheless, we decided to explore also how pre-IVT factors such as plasmid linearization isolation methods could affect to the production of this important impurity. The recent findings regarding COVID have been highly informative, prompting us to investigate whether the purification of linearized plasmids using hydrophobic C4 columns could also reduce the dsRNA content in transcripts of various lengths or sizes. It is well-known that AEX chromatography has proven to be an effective technique for removing dsRNA. However, we aimed to determine if chromatographic purification of DNA templates alone could achieve dsRNA levels below 1% without the need for an additional mRNA polishing step, relying solely on Oligo-dT purification for the transcripts. In this study, our objective was to establish baseline levels of dsRNA generated without the inclusion of supplementary purification steps aimed at its removal or mitigation. To achieve this, we initiated the synthesis of respective mRNA strands for three designated protein targets from their corresponding linearized templates, followed by template purification utilizing the Wizard^®^ Megapreps DNA Purification Resin or chromatographic methodologies involving the CIMmultus^®^ C4HLD matrix. After the purification of templates, an Oligo-dT affinity chromatography process was employed to refine the mRNA constructs, ultimately leading to the acquisition of purified mRNA entities. Subsequently, dsRNA quantification was performed using Dot Blot analysis, as depicted in Figure 4.

**FIGURE 5:**
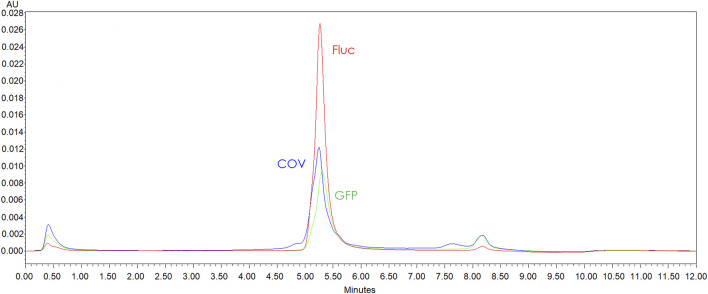
AEX-HPLC (CIMac™ pDNA 0.3 mL Analytical Column (1.4 µm)) profile comparison GFP (green line), Fluc (red line) and COV (blue line) linearized plasmids purified by resin (0.5ug each template injected). Chromatogram shows more intense impurity peaks for COV and GFP.

We observed that the constructs encoding GFP and COV displayed elevated levels of dsRNA, whereas Fluc exhibited more favorable levels around 1%. As previously mentioned, in the absence of a polishing step, the RNA transcripts for GFP and COV exhibited higher dsRNA values compared to Fluc. These findings captured our attention, as the capillary electrophoresis, HPLC, and AGE analyses revealed linearized plasmids targeting COV and GFP with decreased purity and an increased number of peaks that do not correspond with target mRNA. (Figure 5). This observation led us to speculate about a potential relationship between the impurities of the initial template and the production of endogenous dsRNA during IVT.

To assess the impact of linearized template quality, we sought to employ a more specific and reliable purification technique instead of depending solely on simple filtration or resin retention methods. While several options exist for the chromatographic purification of circular plasmids, limited information is available regarding the purification of linearized plasmids. In this study, we investigated the efficacy of CIMmultus C4 HLD columns, which utilize hydrophobic interactions to fractionate proteins from nucleic acids, commonly applied in the polishing of purified mRNA.

To evaluate the performance of C4 columns, we enzymatically opened several circular plasmids of varying lengths using BspQI enzyme, followed by purification using this monolithic column. One example of purification is described in (Supplementary Figure S1). In the supplementary image, the chromatogram depicts a multi-stage process. Initially, during the loading phase, no breakthrough is observed, indicating that all components of the loaded sample (linearized DNA + restriction enzyme) adhere tightly to the column. The elution of the restriction enzyme only occurs after a final treatment involving CIP with 0.1M NaOH. Subsequent to the loading phase, a washing phase with buffer A is employed, followed by elution using a 100% B. This elution process yields a primary peak alongside a minor tail consisting of highly diluted fractions. It is noteworthy that the UV signal within these dilute fraction’s ranges from 20 to 0 mAU. Our extensive experience has shown that the quantity of DNA present in these extremely diluted fractions is negligible compared to the total content of the primary peak, rendering them unsuitable for further analysis and thus necessitating their exclusion. The presence of this tailing-off effect in the chromatogram is attributed to residual components firmly bound to the column, which ultimately detach due to the decreasing conductivity values as the elution progresses. To further investigate these tail fractions, they were consolidated, concentrated, and subjected to preliminary analysis using HPLC CIMac pDNA. This analysis revealed an overlap with the target linear DNA, leading us to conclude that the main peak and the tail were essentially identical (Supplementary Figure S2). However, the mass obtained from these tail fractions constituted less than 5% of the total, prompting their exclusion from further analysis.

**FIGURE 6:**
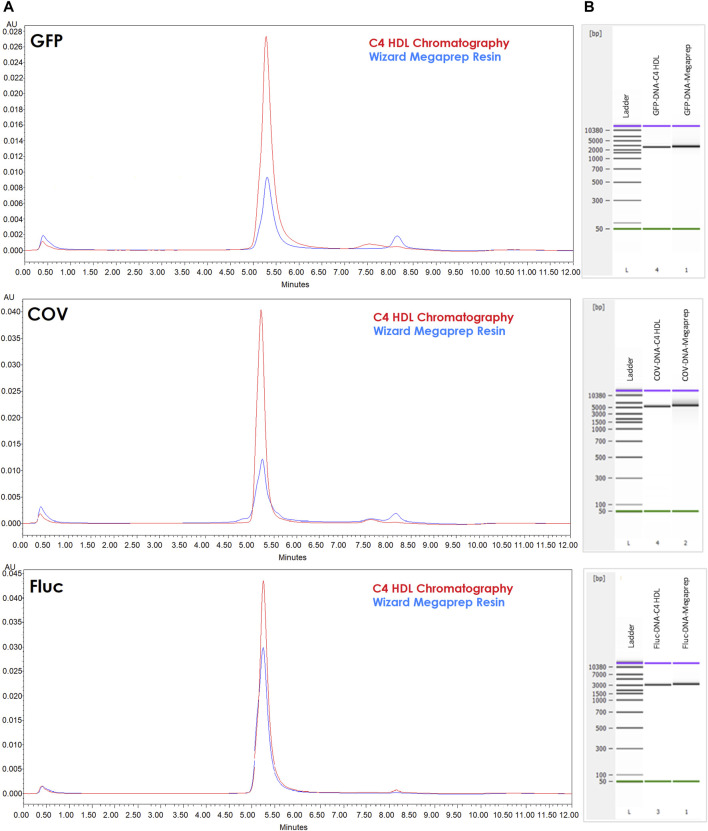
Template characterization. **(A)** AEX-HPLC (CIMac™ pDNA) analytical profile of linear DNA profiles for encoded GFP, Fluc and COV purified by Megapreps DNA Resin (blue lines) and CIMmultus C4 HLD column (red lines) (0.5ug each template injected). **(B)** Capillary electrophoresis for the same samples.

When comparing the purified fractions to templates purified using Megapreps DNA Resin, it became evident that C4 provided superior results. Capillary electrophoresis (CE) profiles exhibited reduced tailing (smearing in the simulated gel) at the right side and higher intensity, indicating improved template purity. Furthermore, HPLC analysis using ClMac™ pDNA demonstrated more prominent main peaks corresponding to the template and decreased impurities (Figure 6).

To further characterize the purified templates, we subjected them to AEX-HPLC and capillary electrophoresis (Figure 6). Comparative analysis revealed improved profiles for the chromatographically purified templates, with reduced degradation and fewer impurity peaks. Notably, the profiles of Fluc templates exhibited greater similarity and lower impurity levels, aligning with the lower dsRNA values observed in the corresponding transcripts.

These results highlight the importance of template purification in obtaining high-quality linearized plasmids. The use of C4 columns offers significant advantages in terms of template integrity and purity, ultimately contributing to the production of mRNA transcripts with reduced levels of impurities, such as dsRNA.

**FIGURE 7:**
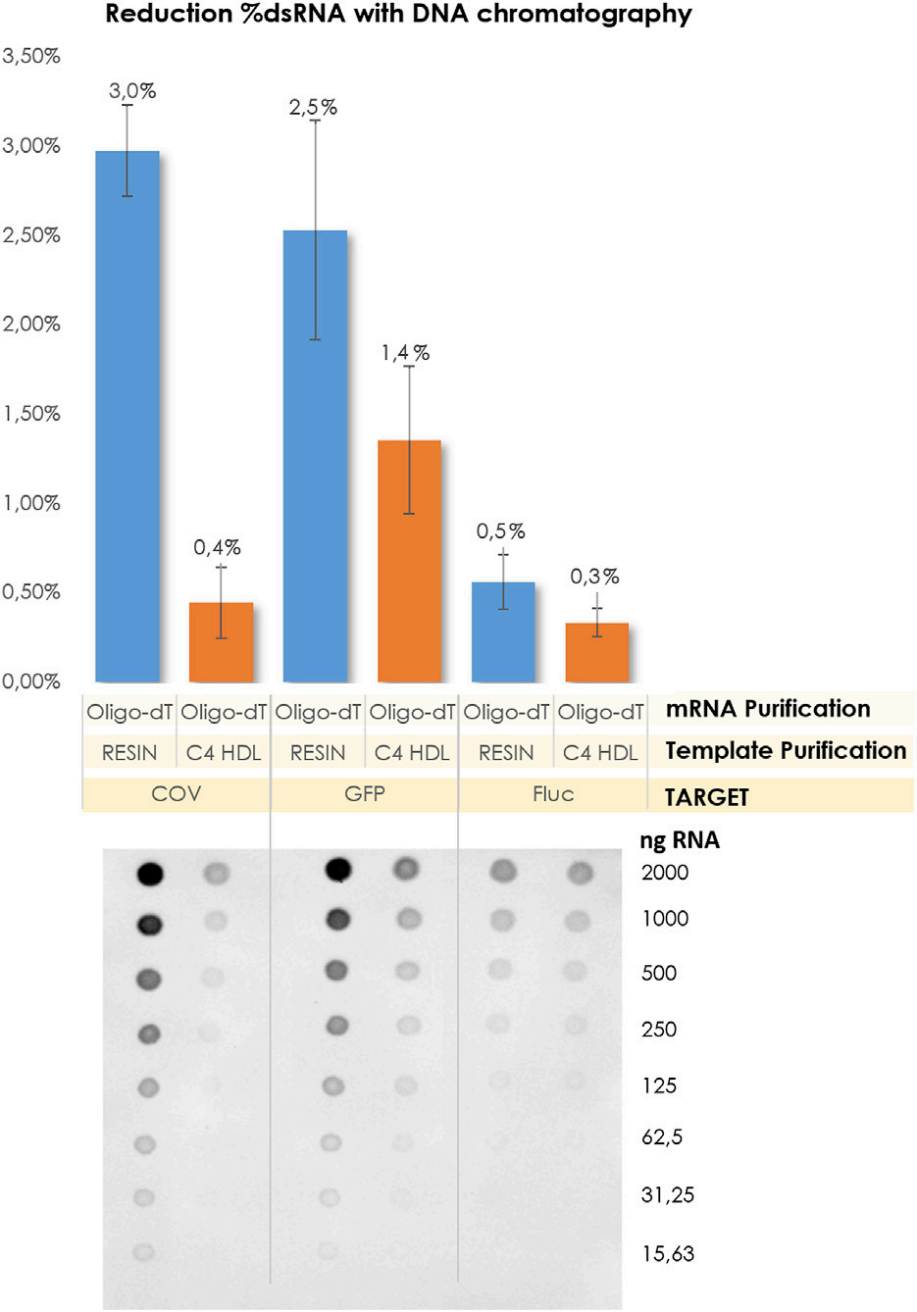
Comparison of dsRNA content related to the template purification method and transcript size. Corresponding dot blot membranes.

The purified linearized plasmids obtained through C4 chromatography were used as the starting material for the subsequent IVT reactions. Following IVT, mRNA crudes were isolated exclusively using affinity chromatography. As anticipated, the levels of dsRNA were significantly reduced, regardless of the size of the resulting mRNA molecules. Notably, the most substantial reduction in dsRNA content was observed for the COVID construct (Figure 7).

To the best of our knowledge, our findings represent the first direct evidence of a correlation between the plasmid purification method and the levels of dsRNA generated during the subsequent IVT process. While it is evident that the use of high-quality linearized plasmids leads to a significant reduction in double-stranded impurities, the underlying explanation for this phenomenon remains unclear.

As previously mentioned, two main mechanisms have been proposed to explain the origin of dsRNA. The first mechanism is associated with the occasional mispriming of the T7 RNA polymerase (T7RNAP) (Triana-Alonso et al., 1995; Gholamalipour et al., 2018; Mu et al., 2018; Mu and Hur, 2021), and the second one in which the antisense strand can be transcribed in a promoter-independent manner, leading to the generation of a complete complementary sequence that anneals to the sense strand (Mu and Hur, 2021) (Figure 1). However, until now, no studies have investigated the influence of impurities on dsRNA generation.

**FIGURE 8:**
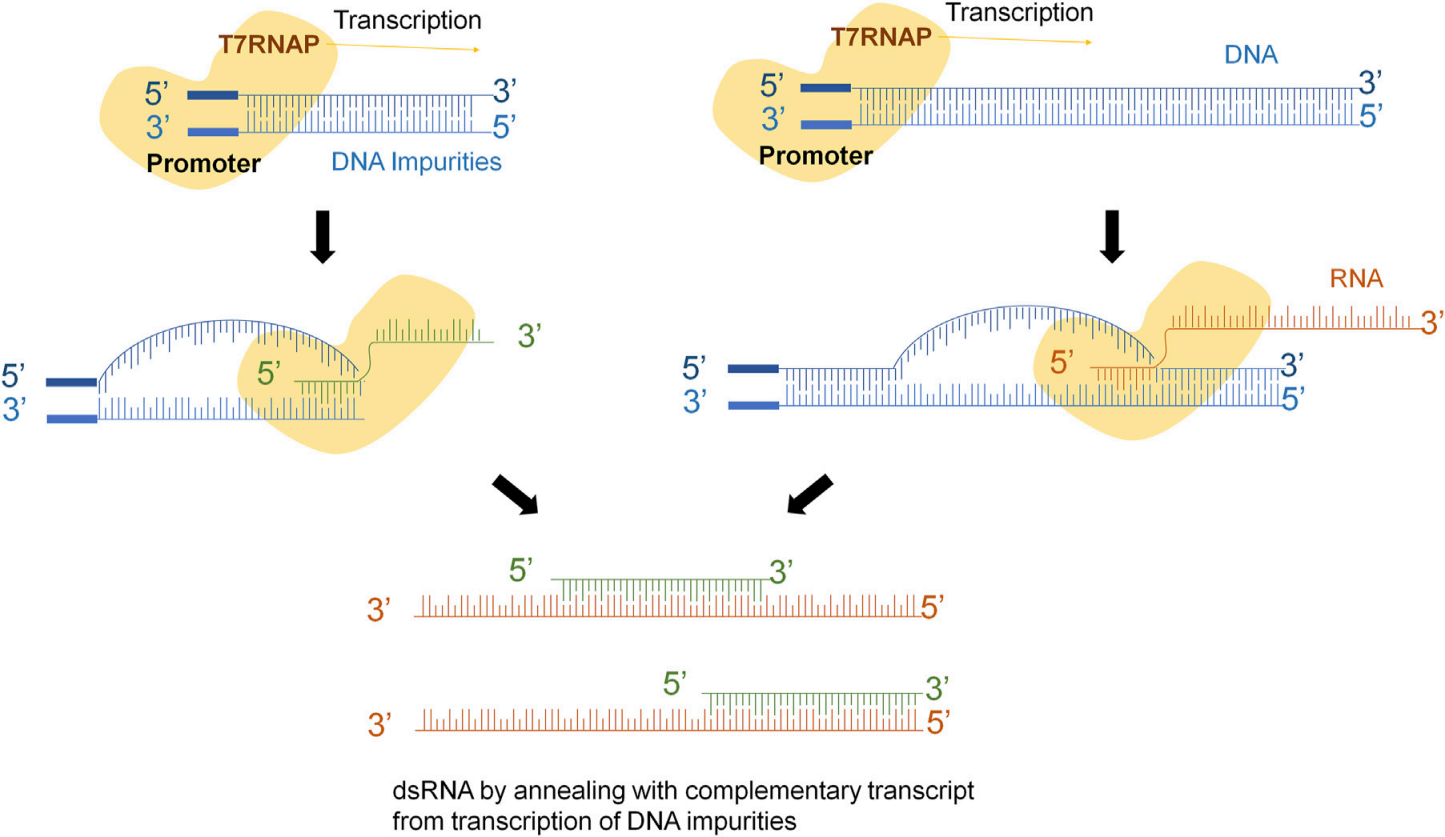
dsRNA generation mechanisms during IVT. Not removed impurities of DNA might be treated as the linearized complete plasmid producing sequences of RNA that could anneal with the original transcript. That might lead to a population of different dsRNA species.

While our study does not aim to elucidate the specific mechanisms or origins of dsRNA, it highlights the critical role of the quality of linearized templates in obtaining transcripts with reduced levels of dsRNA. Importantly, our results suggest that the poor purification of the starting plasmid results in the presence of impurities that the T7 RNAP can potentially use as alternative templates for transcription (Figure 8). Each of these alternative templates could generate RNA fragments that are complementary to different regions of the properly transcribed mRNA sequence. Consequently, a heterogeneous population of dsRNA transcripts would coexist with the dsRNA produced through the aforementioned mechanisms.

The significant reduction of dsRNA observed during the purification of linearized plasmids highlights the substantial contribution of this impurity from the DNA fragments themselves. This finding emphasizes the importance of employing effective purification techniques for linearized templates. The data presented in this study demonstrate the successful application of chromatography, specifically using C4 columns, for this purpose.

In conclusion, our study demonstrates the importance of implementing both purification and polishing steps in the production of mRNA constructs. The use of AEX chromatography proved to be highly effective in reducing dsRNA levels, with some constructs achieving levels below the recommended threshold. However, it was observed that HIC was not as efficient in reducing dsRNA content compared to AEX. The results suggest that by employing a robust purification method for the initial linearized plasmids, such as hydrophobic chromatography, it is possible to obtain mRNA transcripts with lower dsRNA percentages without the need for additional polishing steps. This highlights the importance of optimizing the purification process of the starting linearized plasmid to achieve high-quality mRNA constructs with reduced dsRNA impurities. Further investigations should focus on understanding the underlying mechanisms and factors influencing dsRNA formation, as well as exploring alternative purification strategies for even more efficient removal of dsRNA.

## 4 Materials and methods

### 4.1 Synthetic methods

#### 4.1.1 Plasmid linearization

Prior to RNA IVTs, pDNAs encoding firefly luciferase (pTLuc), spike 2p SARS-CoV-2 (pTCOV01), and green fluorescent protein (pTGFP) were linearized using BspQI (NEB) enzyme, according to the manufacturer’s instructions.

#### 4.1.2 IVT mRNA synthesis

Triphosphate-derivatives of *N*-methylpseudouridine (Hongene) were used to generate modified nucleoside containing RNA. All RNAs were capped using Cleancap™ (Trilink Biotechnologies). All IVT reagents except enzymes listed in (Table 1) were preheated to 37°C, mixed in a 1.5 mL plastic tube in Thermomixer^TM^C (Eppendorf) and incubated at 37°C with shaking at 300 rpm. The reaction was quenched after 180 min by addition of 5 mM EDTA, pH 8.0 for its subsequent purification by the different methods.

**TABLE 1:** Components for upstream production.

Synthesis step	Component	Reaction conditions
Template DNA Linearization	Plasmid	50°C for 1 h
	BspQI enzyme	
	10X Dig. buffer	
	Nuclease free water	
Transcription (IVT)	NTPs (ATP, CTP, GTP)	37°C for 3 h
	N-Methyl-Pseudouridine	
	Linearized plasmid	
	CleanCap™	
	10X IVT buffer	
	T7 RNAP	
	Pyrophosphatase	
	RNAse inhibitor	
	Nuclease free water	
Template DNA Digestion	DNase	37°C for 15 min
	10X Dnase buffer	
Quenching	EDTA	N/A

### 4.2 Purification methods

#### 4.2.1 Purification of linearized plasmids with silica resin

The Wizard^®^ Plus Maxipreps DNA Purification System provides a rapid resin-based batch column method for purification of plasmid DNA. The purification procedure was carried out following the manufacturer’s protocol.

#### 4.2.2 Hydrophobic interaction chromatography of linearized plasmids

Chromatographic purification was performed on the ÄKTA Start (GE Healthcare) FPLC system composed of two pumps and a multiwavelength UV-Vis detector (2 mm flow cell path length). Unicorn software (GE Healthcare) was used for instrument control and data acquisition. DNA template linearized was diluted once in sample loading buffer (75 mM Tris + 15 mM EDTA + 3.75 M SA (ammonium sulphate) pH = 7.2 and loaded onto CIMmultus C4 HDL 1 mL column (Sartorius) equilibrated in mobile phase containing 50 mM Tris + 10 mM EDTA + 2.5 M SA pH = 7.2. After the UV 260 nm signal and conductivity was stabilized, an elution step was performed with 50 mM Tris + 10 mM EDTA pH = 7.2 DNA content from desired fractions was concentrated and desalted using Amicon Ultra-15 centrifugal filter units (30K membrane) (Millipore) by successive centrifugation at 1,000 g for 10 min RT in a 5804R centrifuge (Eppendorf).

#### 4.2.3 Purification of mRNA by lithium chloride precipitation

RNA precipitation is an easy and cost-effective method for the concentration of RNA, leaving a pellet that can be resuspended in the buffer of choice. An equal volume of LiCl 7.5 M solution was added, and the resulting solution was stored at −80°C overnight. Next day, it was centrifuged at top speed for 20 min and the supernatant was discarded. The pellet was washed twice with ice-cold 70% ethanol to remove residual salt and dried with SpeedVac for 5 min. The pellet was then resuspended in a suitable buffer and stored at −80°C.

#### 4.2.4 Affinity chromatography of mRNA

The ssRNA fraction from CIMmultus PrimaS was purified by affinity chromatography using POROS Oligo (dT)25 (ThermoFisher) as polishing step.

Buffer A contained 50 mM sodium phosphate, 0.5 M NaCl, 5 mM EDTA, pH = 7.0 and Buffer B contained 50 mM sodium phosphate, 5 mM EDTA, pH = 7.0. Column was equilibrated with 100% Buffer A, loaded with RNA, washed with buffer B, and finally eluted by step elution of polyadenylated mRNA with double-deionized water.

#### 4.2.5 RNA isolation from column fractions

RNA content from desired fractions was concentrated and desalted using Amicon Ultra-15 centrifugal filter units (30K membrane) (Millipore) by successive centrifugation at 2,100 g for 10 min in a 5804R centrifuge (Eppendorf), and dilution with nuclease free water.

#### 4.2.6 Anion exchange chromatography of mRNA

CIMmultus PrimaS is used for one-step purification of research grade of ssRNA, to process *in vitro* transcription mixtures. Purification performance is enhanced by a high-salt wash that removes the majority of dsRNA, DNA, and proteins in advance of elution. IVT mixture was diluted once in sample loading/equilibrate buffer A (20 mM Tris, 20 mM BTP, 20 mM glycine, 50 mM NaCl, 10 mM EDTA, pH = 8.0) and loaded onto CIMmultus PrimaS (BIA Separations). After unbound IVT components eluted in flow-through, a wash 1 with equilibration buffer was necessary, followed by a high-salt wash with 50 mM Tris, 3.0 M guanidine-HCl, 20 mM EDTA, pH 8.0. DNA, dsRNA, and proteins typically elute on the ascending side of the guanidine-EDTA peak. And after a wash 3 with buffer A until UV returns to baseline, a step elution was performed with buffer C (20 mM Tris, 20 mM BTP, 20 mM glycine, 50 mM NaCl, 10 mM EDTA, pH = 11.0). Fractions were neutralized immediately after elution.

### 4.3 Analytical methods

#### 4.3.1 HPLC analysis of pDNA

Linear pDNA was analyzed by HPLC using a CIMac^TM^ pDNA-0.3 Analytical Column (Pores 1.4 µm) (BIA Separations) connected to an HPLC WATERS e2695.

The separations of the isoforms were performed in a linear gradient of NaCl with the temperature of the whole system controlled to 15.0+/- 0.5°C. Using the followed mobile phases in standard protocol for the column (MPA: 100 mM Tris-HCl, 0.6 M NaCl, pH = 8, MPB: 100 mM Tris-HCl, 1 M NaCl, pH = 8). A linear gradient was performed, 5% MPB to 30% MPB in 6 min at a flow rate 1 mL/min. The column was eluted with 100% MPB for 1 min before re-equilibration in 5% MPB.

#### 4.3.2 Dot blot

Purified IVT RNA samples were spotted onto positively charged nylon membranes (Nytran SC, Sigma Aldrich). The membranes were blocked with 5% (w/v) non-fat dried milk in TBS-T buffer (20 mM Tris-HCl, 150 mM NaCl, 0.1% [v/v] Tween-20, pH 7.4), and incubated with dsRNA-specific mAb J2 (Jena Bioscience) 4°C ON. Membranes were washed three times with TBS-T and reacted with HRP-conjugated goat anti-mouse Ig (Abcam), washed three times, and detected with ECL Plus Western blot detection reagent (Amersham). Images were captured on an iBright 750 digital imaging system.

dsRNA Ladder (Biolabs) (25 ng) was used as a positive control.

#### 4.3.3 Capillary electrophoresis

DNA and RNA samples were loaded into the corresponding kits; Agilent DNA 12000 Kit and Agilent RNA 6000 Nano Kit, both designed for use with the Agilent 2,100 Bioanalyzer instrument, according to guide instructions. Results were analyzed with the 2,100 Expert Software.

## Data Availability

The raw data supporting the conclusion of this article will be made available by the authors, without undue reservation.
