# Supplementary material for: Purification of linearized template plasmid DNA decreases double-stranded RNA formation during IVT reaction
Source: Front Mol Biosci. 2023 Sep 29;10:1248511. doi: 10.3389/fmolb.2023.1248511 (PMC10570549; doi:10.3389/fmolb.2023.1248511)
Supplement: Supplementary file 1 [file Table1.DOCX]

Supplementary Material

# Supplementary Figures and Tables

##
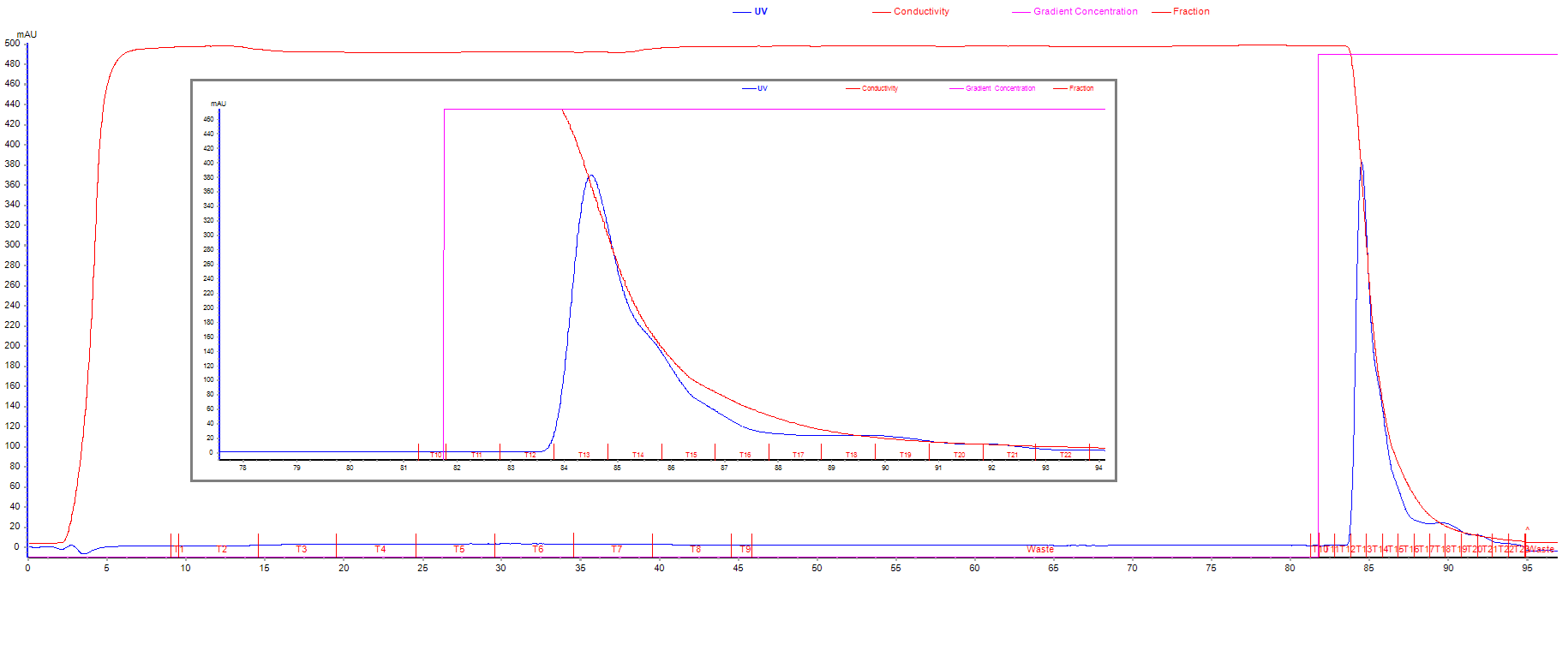
Supplementary Figures

**Supplementary Figure 1.** Purification chromatogram of COV linear plasmid. Chromatographic purification was performed on the ÄKTA Start (GE Healthcare) FPLC system composed of two pumps and a multiwavelength UV-Vis detector (2 mm flow cell path length). Unicorn software (GE Healthcare) was used for instrument control and data acquisition. 20 ml of crude DNA linearized template (≈1000 µg) was diluted once in 37 mL of sample loading buffer (75 mM Tris + 15 mM EDTA + 3.75 M SA (ammonium sulphate) pH = 7.2 and loaded onto CIMmultus C4 HDL 1 mL column (Sartorius) equilibrated in mobile phase containing 50 mM Tris + 10 mM EDTA + 2.5 M SA pH = 7.2. After the UV 260 nm signal and conductivity was stabilized, an elution step was performed with 50 mM Tris + 10 mM EDTA pH = 7.2. Desired fractions (T12-T16 fractions) were concentrated and desalted using Amicon Ultra-15 centrifugal filter units (30K membrane) (Millipore) by successive centrifugation at 1000 g for 10 min RT in a 5804R centrifuge (Eppendorf).

*
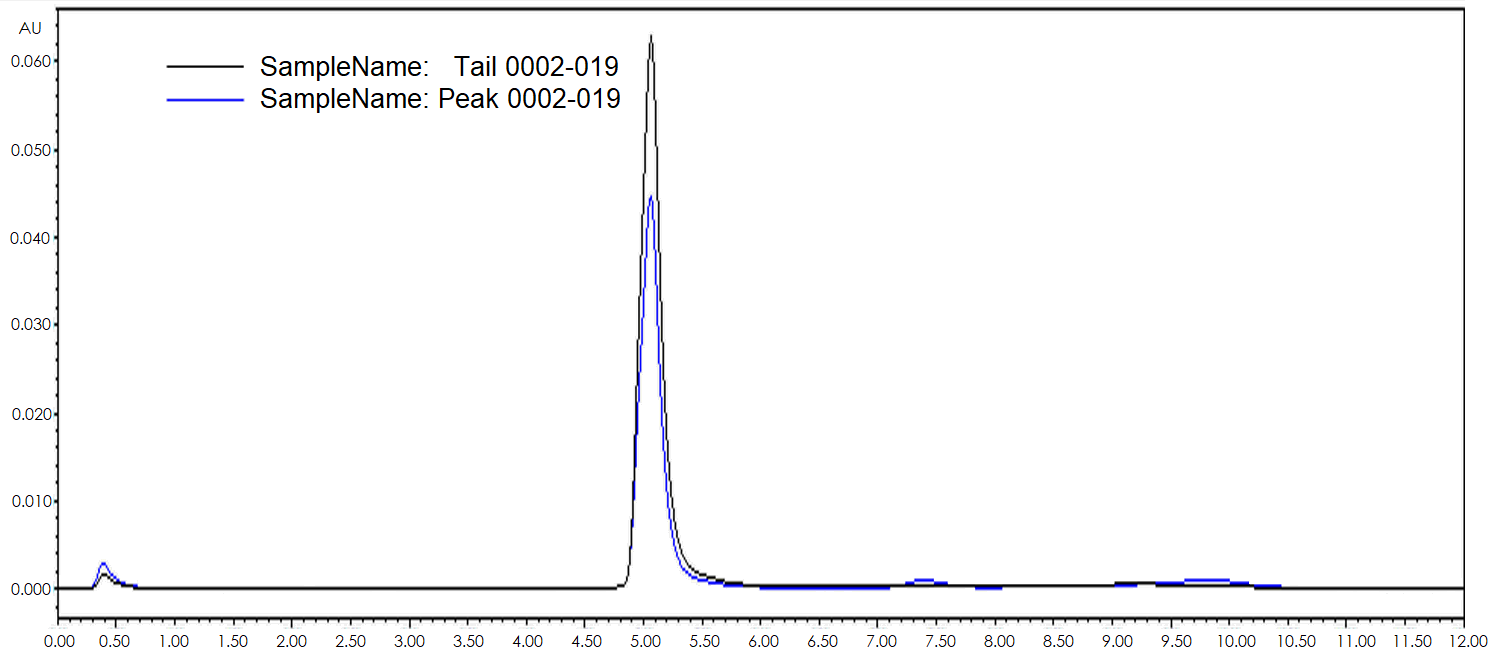
*

**Supplementary Figure 2.** Comparison of analytical chromatogram of mail peak fractions of COV linear plasmid (blue line) *vs* concentrated tail fractions (black line). No significant differences between both profiles are found. It can be consider thar main peak and tail fractions has not content any additional impurity.
