# Supplementary material for: Purification of linearized template plasmid DNA decreases double-stranded RNA formation during IVT reaction
Source: Front Mol Biosci. 2023 Sep 29;10:1248511. doi: 10.3389/fmolb.2023.1248511 (PMC10570549; doi:10.3389/fmolb.2023.1248511)
Supplement: Supplementary file 2 [file DataSheet1.PDF]

Supplementary Material

2 Supplementary Figures and Tables

2.1 Supplementary Figures

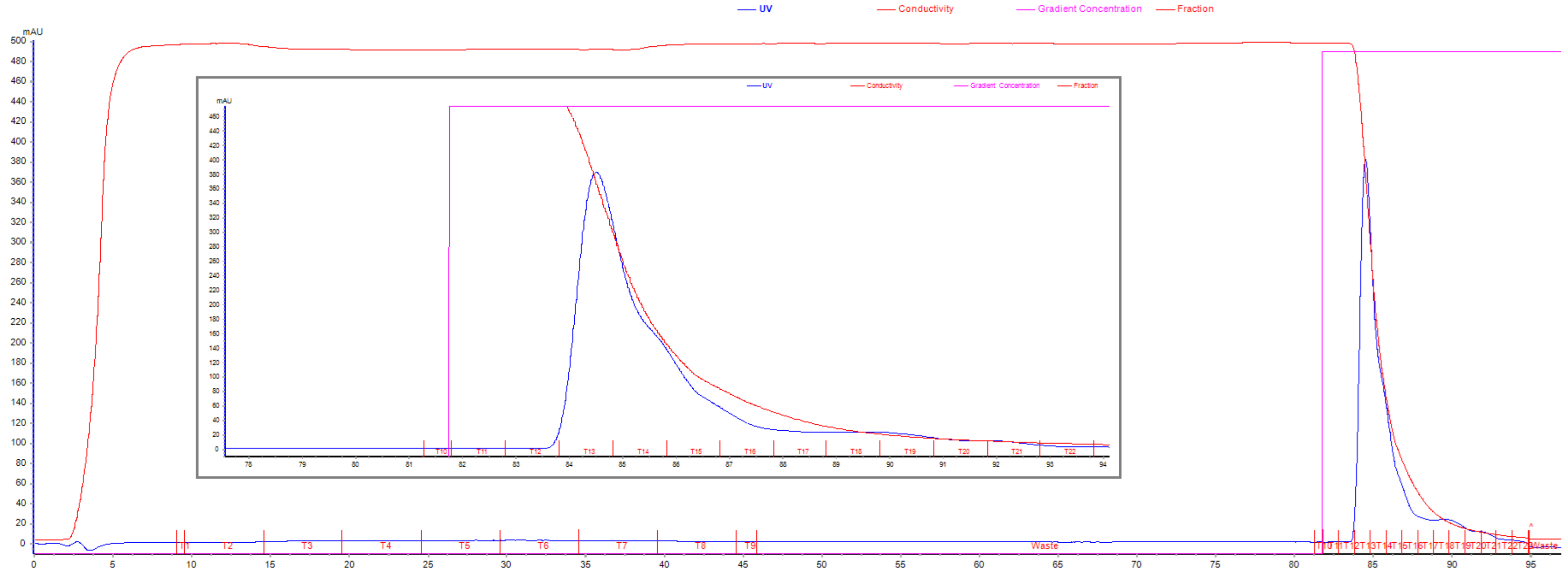

Supplementary Figure 1. Purification chromatogram of COV linear plasmid. Chromatographic purification was performed on the ÄKTA Start (GE Healthcare) FPLC system composed of two pumps and a multiwavelength UV-Vis detector (2 mm flow cell path length). Unicorn software (GE Healthcare) was used for instrument control and data acquisition. 20 ml of crude DNA linearized template (≈1000 µg) was diluted once in 37 mL of sample loading buffer (75 mM Tris + 15 mM EDTA + 3.75 M SA (ammonium sulphate) pH = 7.2 and loaded onto CIMmultus C4 HDL 1 mL column (Sartorius) equilibrated in mobile phase containing 50 mM Tris + 10 mM EDTA + 2.5 M SA pH = 7.2. After the UV 260 nm signal and conductivity was stabilized, an elution step was performed with 50 mM Tris + 10 mM EDTA pH = 7.2. Desired fractions (T12-T16 fractions) were concentrated and desalted using Amicon Ultra-15 centrifugal filter units (30K membrane) (Millipore) by successive centrifugation at 1000 g for 10 min RT in a 5804R centrifuge (Eppendorf).

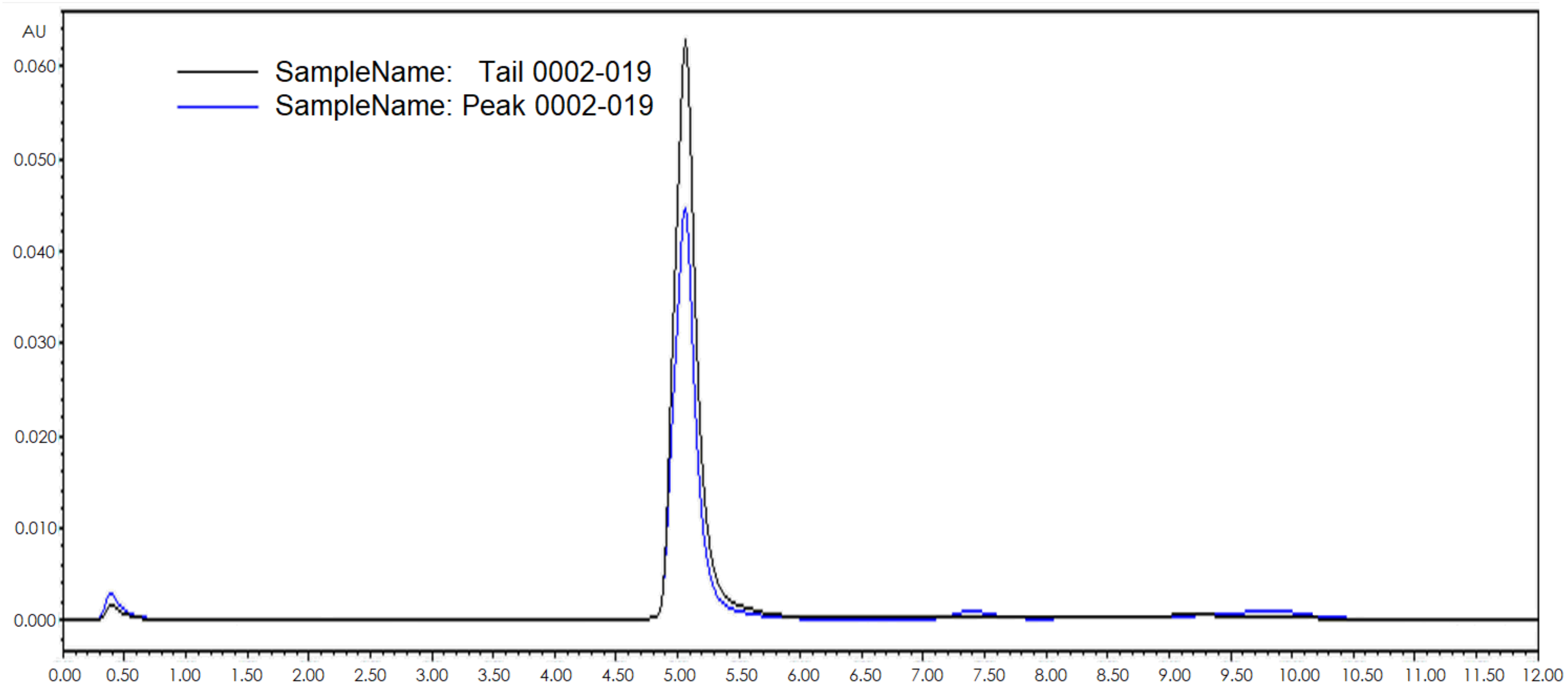

Supplementary Figure 2. Comparison of analytical chromatogram of mail peak fractions of COV linear plasmid (blue line) *vs* concentrated tail fractions (black line). No significant differences between both profiles are found. It can be consider thar main peak and tail fractions has not content any additional impurity.
